# Upadacitinib for Treatment of Granulomatous Cheilitis

**DOI:** 10.1001/jamadermatol.2024.2378

**Published:** 2024-07-31

**Authors:** Axel De Greef, Caroline Peeters, Olivier Dewit, Laurence de Montjoye, Marie Baeck

**Affiliations:** 1 Department of Dermatology, Cliniques universitaires Saint-Luc, Université catholique de Louvain (UCLouvain), Brussels, Belgium; 2 Institute of Experimental and Clinical Research (IREC), Pneumology, ENT and Dermatology Pole (LUNS), Université catholique de Louvain (UCLouvain), Brussels, Belgium; 3 Department of Gastroenterology, Cliniques universitaires Saint-Luc, Université catholique de Louvain (UCLouvain), Brussels, Belgium; 4 Institute of Experimental and Clinical Research (IREC), Pole of Research in Hepato-Gastroenterology (GAEN), Université catholique de Louvain (UCLouvain), Brussels, Belgium

## Abstract

This case series examines the effectiveness of the Janus kinase inhibitor upadacitinib for the treatment of granulomatous cheilitis.

Granulomatous cheilitis (of Miescher) (GC), a rare nonnecrotizing granulomatous inflammatory disorder, is part of the orofacial granulomatosis spectrum.^1,2^ It is characterized by intermittent or persistent swelling of the upper and/or lower lip, resulting in functional disability and cosmetic impairment. Despite various hypotheses, including factors such as food allergy, genetics, infection, and atopy, its exact cause remains unknown.^2^ Granulomatous cheilitis is an exclusion diagnosis, and most cases remain isolated; however, it can be associated with systemic diseases such as Crohn disease (CD),^3^ sarcoidosis, or Melkersson-Rosenthal syndrome. Current treatment primarily involves topical or intralesional corticosteroids, with severe cases often requiring systemic agents such as biologics, with variable outcomes and frequent relapses.^4^ The evidenced role of the Janus kinase–signal transducer and activator of transcription (JAK-STAT) pathway in granuloma formation has brought interest in using JAK inhibitors for granulomatous skin diseases^5^; to our knowledge, their effectiveness in GC remains unexplored.

## Methods

This retrospective case series was conducted from June 1, 2023, to March 1, 2024, at a tertiary university hospital in Belgium, involving patients with biopsy-proven GC resistant to systemic treatments and subsequently treated with the JAK 1 inhibitor upadacitinib, 30 mg daily. All patients underwent standardized diagnostic work-up with complete cutaneous and extracutaneous examination, lip biopsy, chest radiograph, and colonoscopy. Objective clinical improvement in lip swelling and infiltration was assessed, with either complete response, partial response, or no response as the primary end point. Effectiveness on CD activity in patients with concomitant GC and CD was the secondary end point. All treatment-induced adverse events were reported during the study. We followed the reporting guideline for case series. The study and data collection were approved by the institutional review boards of Cliniques universitaires Saint-Luc and Université catholique de Louvain. Written informed consent was obtained from all study participants for publication of their case details.

## Results

Five patients (4 women; median age, 30 years [range, 20-48 years]) were included. Demographic and clinical characteristics are detailed in the Table. All patients were previously treated with biologics in an attempt to control GC, CD, or both. Three patients had concomitant quiescent CD. Upadacitinib was added, but ustekinumab was maintained for patient 5 because of history of very severe CD with coloprotectomy.

**Table.** Patients’ Demographic and Clinical Characteristics and Responses to Upadacitinib Treatment

Patient No./sex/age, decade	Self-reported ethnicity	Duration of GC, y	Comorbidities	Histopathologic findings	Workup	Previous treatments for GC (excluding biologics)	Previous biologics^a^	Response with upadacinitib, 30 mg	Follow-up duration	Adverse events, TTO
1/F/30s	White	10	None	Epithelioid nonnecrotizing granulomas with perivascular distribution (inferior lip biopsy)	Normal physical examination results, normal chest radiograph results, negative colonoscopy for IBD^b^	Intralesional CS, systemic CS, methotrexate, dapsone	Infliximab (15 mo); guselkumab, 100 mg/4 wk (9 mo); risankizumab, 300 mg/8 wk (8 mo)	GC: CR after 4 mo	7.2 mo	Acne, mild headaches (2 mo); TC, 213 mg/dL; LDL-C, 128 mg/dL (5 mo)
2/F/20s	Arab	13	CD (CDAI at inclusion = 80)	Numerous epithelioid nonnecrotizing granulomas (inferior lip biopsy)	Normal physical examination results, normal chest radiograph results, positive colonoscopy at CD diagnosis^b^	Intralesional CS, systemic CS, tetracycline, disulone	Infliximab (26 mo); risankizumab, 300 mg/8 wk (16 mo)	GC: PR–slight permanent noninfiltrated swelling of the lip CD: CDAI = 92, endoscopically quiescent	7.9 mo	ALT, 44 U/L (4 mo)
3/M/20s	Arab	11	CD (CDAI at inclusion = 102), atopic dermatitis	Numerous epithelioid nonnecrotizing granulomas (superior lip biopsy)	Normal physical examination results, normal chest radiograph results, positive colonoscopy at CD diagnosis^b^	Topical CS, intralesional CS, metronidazole, colchicine, systemic CS, tetracycline, isotretinoin, disulone, azathioprine	Adalimumab (31 mo); risankizumab, 300 mg/8 wk (16 mo)	GC: CR after 3.1 mo CD: CDAI = 113; fecal calprotectin, 30 μg/g (NV, 0-50)	6.3 mo	None
4/F/40s	White	2	Melkerson-Rosenthal syndrome, quiescent pancolitis, MGUS IgG κ	Epithelioid nonnecrotizing granulomas (superior lip biopsy)	Normal physical examination results, normal chest radiograph results, positive colonoscopy at pancolitis diagnosis^b^	Intralesional CS, systemic CS	Guselkumab ≤200 mg/4 wk (8 mo)	GC: CR after 5 mo	7.4 mo	None
5/F/30s	White	5	CD (normal fecal calprotectin, 22 μg/g, CDAI at inclusion not available)	Epithelioid nonnecrotizing granulomas (superior lip biopsy)	Normal physical examination results, normal chest radiograph results, positive colonoscopy at CD diagnosis^b^	Tetracycline	Ustekinumab, 90 mg/4 wk (62 mo)	GC: CR after 3.6 mo CD: fecal calprotectin, 22 μg/g (NV, 0-50)	3.6 mo	None

Abbreviations: ALT, alanine aminotransferase; CD, Crohn disease; CDAI, Crohn’s Disease Activity Index; CR, complete response; CS, corticosteroids; GC, granulomatous cheilitis; IBD, inflammatory bowel disease; IgG, immunoglobulin G; LDL-C, low-density lipoprotein cholesterol; MGUS, monoclonal gammopathy of undetermined significance; NV, normal value; PR, partial response; TC, total cholesterol; TTO, time to onset.

SI conversion factor: To convert total cholesterol and LDL-C to millimoles per liter, multiply by 0.0259; and ALT to microkatals per liter, multiply by 0.0167.

^a^ Biologics that failed to treat either GC, CD, or both.

^b^ Normal cutaneous (except the lips) and extracutaneous examination.

The median follow-up duration was 7.2 months (range, 3.6-7.9 months). Upadacitinib treatment was associated with complete response in 4 of 5 patients (80%) within a median of 3.8 months (range, 3.1-5.0 months) (Figure); 1 patient showed partial response. Crohn disease remained quiescent in 3 of 3 patients with concomitant GC and CD. The treatment was well tolerated with no serious adverse events reported. Headaches, acne, mild lipid profile modifications, and/or transaminitis were observed in 2 patients.

**Figure.**
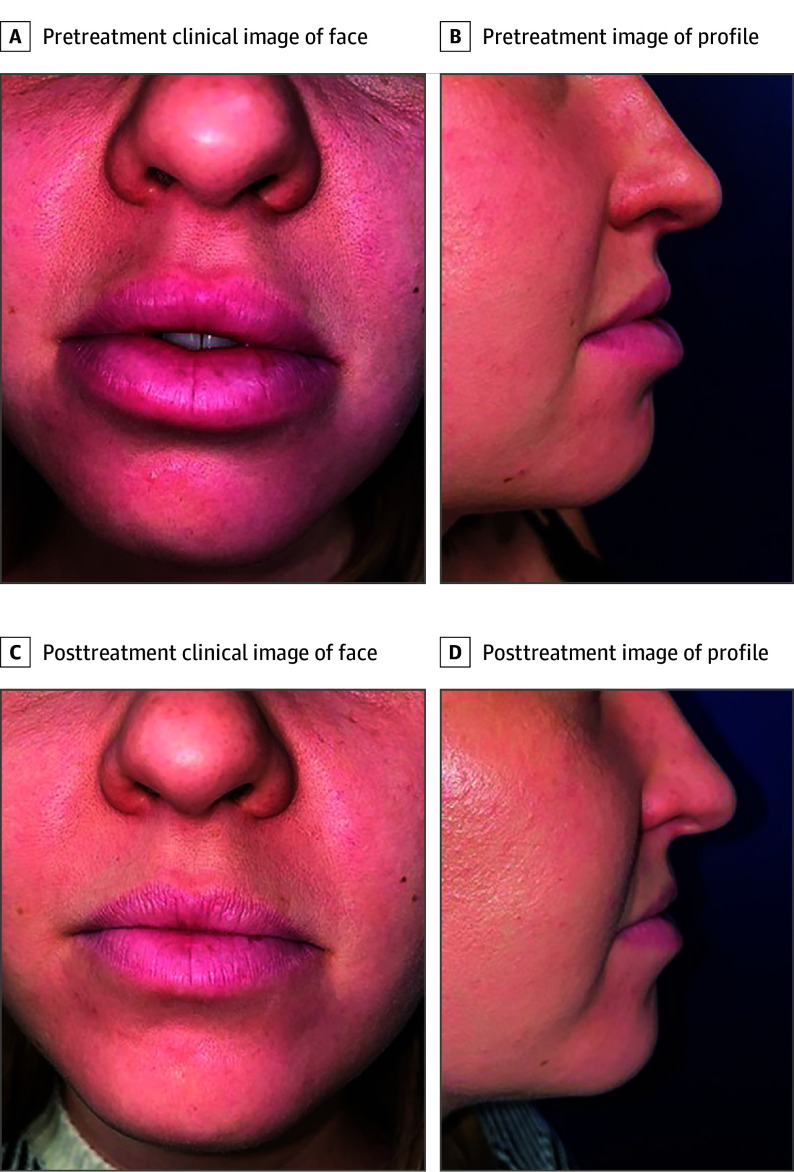
Clinical Evolution of Patient 1 With Upadacitinib Clinical aspect of granulomatous cheilitis before (A) and after (B) 4 months of treatment with upadacitinib, 30 mg.

## Discussion

JAK inhibitors have emerged as promising therapeutic options for various inflammatory disorders, including granulomatous diseases.^5^ Granulomatous cheilitis is a rare and debilitating granulomatous disorder that often represents a therapeutic challenge. All 5 patients in our study with longstanding recalcitrant GC showed meaningful clinical response within a median follow-up of 7.2 months when treated with high-dose (30 mg/d) upadacitinib. Crohn disease remained quiescent in 3 of 3 patients with concomitant GC and CD, despite discontinuing biologic treatment in 2 patients and despite a lower-dose regimen of upadacitinib than the one recommended for CD.^6^ The safety profile was favorable, with no serious adverse events. Combining a biologic and upadacitinib for 1 patient was effective and well tolerated.

Limitations of our study are the small sample size and the short follow-up duration, which may limit generalization of the findings to the broader population of patients with GC. Upadacitinib was effective in treating patients with recalcitrant and long-lasting granulomatous cheilitis, even in cases of concomitant CD, which could substantially improve the quality of life of affected patients. Safety data are reassuring. Further studies are needed to confirm these data in larger cohorts, over longer periods, and with other JAK inhibitors.

## Notes

Supplement.
Data Sharing Statement
