# Supplementary material for: Upadacitinib for Treatment of Granulomatous Cheilitis
Source: JAMA Dermatol. 2024 Jul 31;160(9):1001–3. doi: 10.1001/jamadermatol.2024.2378 (PMC11292563; doi:10.1001/jamadermatol.2024.2378)
Supplement: Supplement. — Data Sharing Statement [file jamadermatol-e242378-s001.pdf]

Data Sharing Statement

De Greef. Upadacitinib for Treatment of Granulomatous Cheilitis. *JAMA Dermatol*. Published July 31, 2024. doi:10.1001/jamadermatol.2024.2378

Data
Data available: No

Additional Information
Explanation for why data not available: Due to the nature of the research and ethical restrictions, supporting data is not available.
